# Supplementary material for: Association between oral microbiome and seven types of cancers in East Asian population: a two-sample Mendelian randomization analysis
Source: Front Mol Biosci. 2023 Nov 21;10:1327893. doi: 10.3389/fmolb.2023.1327893 (PMC10702768; doi:10.3389/fmolb.2023.1327893)
Supplement: Supplementary file 1 [file DataSheet1.ZIP › Supplementary Materials/MR plots of saliva/saliva═╝/colorectal cancer/pheno.520_to_colorectal cancer_leave_one_out.pdf]

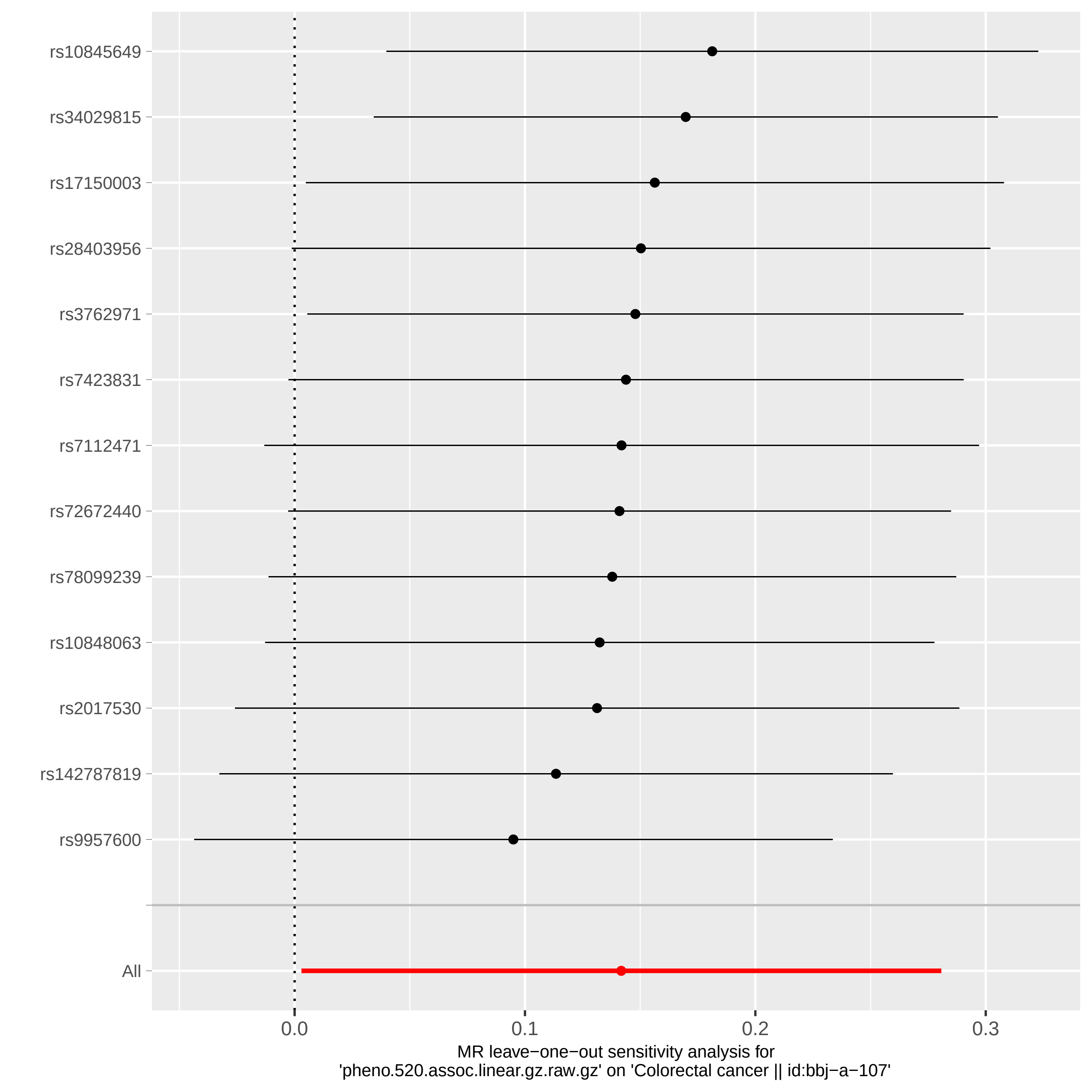
rs10845649
rs34029815
rs17150003
rs28403956
rs3762971
rs7423831
rs7112471
rs72672440
rs78099239
rs10848063
rs2017530
rs142787819
rs9957600
All
0.0
0.1
0.2
0.3
MR leave−one−out sensitivity analysis for
'pheno.520.assoc.linear.gz.raw.gz' on 'Colorectal cancer || id:bbj−a−107'
